# Isolation and Characterization of a Lytic Bacteriophage RH-42-1 of *Erwinia amylovora* from Orchard Soil in China

**DOI:** 10.3390/v16040509

**Published:** 2024-03-26

**Authors:** Haishen Xi, Benzhong Fu, Qiang Sheng, Ming Luo, Liying Sun

**Affiliations:** 1 The Department of Plant Pathology, College of Agronomy at Xinjiang Agricultural University/Key Laboratory of Detection and Control of Agricultural and Forest Pests, Urumqi 830052, China; x990113@sina.com (H.X.); benzhongf@yahoo.com (B.F.); 2 Ministry of Agriculture and Rural Affairs Key Laboratory for Pests and Diseases Control of Northwest Arid Oasis Agricultural Foreign Invasion Species, Urumqi 830052, China; 3 Xinjiang Bayingolin Mongolian Autonomous Prefecture Academy of Agricultural Sciences, Korla 841003, China; xjssqq@sina.com; 4 The Department of Plant Pathology, College of Plant Protection, Northwest A&F University, Yangling 712100, China

**Keywords:** fire blight, *Erwinia amylovora*, bacteriophage, *Alphatectivirus*, genome, resistance, orchard soil, RH-42-1

## Abstract

Fire blight, caused by the bacterium *Erwinia amylovora*, is a major threat to pear production worldwide. Bacteriophages, viruses that infect bacteria, are a promising alternative to antibiotics for controlling fire blight. In this study, we isolated a novel bacteriophage, RH-42-1, from Xinjiang, China. We characterized its biological properties, including host range, plaque morphology, infection dynamics, stability, and sensitivity to various chemicals. RH-42-1 infected several *E. amylovora* strains but not all. It produced clear, uniform plaques and exhibited optimal infectivity at a multiplicity of infection (MOI) of 1, reaching a high titer of 9.6 × 10^9^ plaque-forming units (PFU)/mL. The bacteriophage had a short latent period (10 min), a burst size of 207 PFU/cell, and followed a sigmoidal one-step growth curve. It was stable at temperatures up to 60 °C but declined rapidly at higher temperatures. RH-42-1 remained viable within a pH range of 5 to 9 and was sensitive to extreme pH values. The bacteriophage demonstrates sustained activity upon exposure to ultraviolet radiation for 60 min, albeit with a marginal reduction. In our assays, it exhibited a certain level of resistance to 5% chloroform (CHCl_3_), 5% isopropanol (C_3_H_8_O), and 3% hydrogen peroxide (H_2_O_2_), which had little effect on its activity, whereas it showed sensitivity to 75% ethanol (C_2_H_5_OH). Electron microscopy revealed that RH-42-1 has a tadpole-shaped morphology. Its genome size is 14,942 bp with a GC content of 48.19%. Based on these characteristics, RH-42-1 was identified as a member of the *Tectiviridae* family, *Alphatectivirus* genus. This is the first report of a bacteriophage in this genus with activity against *E. amylovora*.

## 1. Introduction

Fire blight is a quarantine bacterial disease caused by *Erwinia amylovora* in China. It infects fruit trees such as pear, apple, hawthorn, and other species in the *Rosaceae* family [1]. The pathogen invades the host plant’s above-ground tissues, including flowers, young branches, trunk, main stem, fruits, and rootstock, through wounds or natural openings. In severe outbreaks, it can rapidly spread from affected young organs to main branches and stems down to the roots within a few weeks. This can lead to the death of the entire plant, resulting in orchard destruction and substantial economic losses [2]. Fire blight first occurred in Yili and Bazhou, Xinjiang, China in 2016 and 2017, it rapidly escalating and posing a significant threat and risk to the national pear industry [3].

The global fight against fire blight faces a major hurdle due to the scarcity of resistant pear varieties. This necessitates the development of safe and effective prevention and control strategies [4]. Chemical control remains the primary measure for the management of the disease. However, the number of specialized registered chemical agents with significant efficacy against fire blight is limited. Sole reliance on these treatments not only offers limited effectiveness but also contributes to increased antibiotic resistance in the pathogen, pesticide residues, and environmental pollution [5].

In recent years, the biological control of fire blight has made some progress. Biological control agents, such as the fluorescent *pseudomonad* “Blight Ban A506” [6], the broad-spectrum *Pantoea* sp. formulation “Blight BanC9-1” [7], the *Bacillus subtilis* QST713 formulation “Serenade” [8], and BD170 [9] have been applied on a certain scale, showing comparable efficacy to antibiotics under ideal conditions. In China, a group of antagonistic strains exhibiting good activity against fire blight bacteria has been screened, but they are still in the research or trial phase and have not been registered for use [10].

Lytic bacteriophages are a class of viruses that lyse and kill target bacteria. These viruses possess distinct advantages, including strong specificity, rapid proliferation, high efficacy, and resistance prevention [11]. Therefore, their unique bactericidal properties can effectively combat antibiotic-resistant bacterial infections. The utilization of bacteriophages has shown promising results in controlling diseases such as tomato bacterial wilt (*Ralstonia solanacearum*), bacterial speck of tomato (*Pseudomonas syringae* pv. *tomato*), citrus canker (*Xanthomonas citri* subsp. *citri*), and potato soft rot (*Pectobacterium carotovora* subsp. *carotovora*), indicating their potential as alternatives to antibiotic-based antimicrobial agents [12,13].

Research on using bacteriophages to control fire blight disease in pears has made notable progress outside of China. Akremi et al. [14] identified four *E. amylovora* bacteriophages, PEar1, PEar2, PEar4, and PEar6, whose individual or combined use significantly reduced the survival of *E. amylovora*, lowering the incidence of fire blight disease. In 2019, the United States Environmental Protection Agency approved AgriPhageTm-Fire Blight, a bacteriophage-based pesticide for fire blight disease control. However, in China, research in this field is limited, with few reports available [3,15].

In response to fire blight outbreaks in Xinjiang pear orchards, this study aimed to identify potential biocontrol agents. We obtained diverse *E. amylovora* strains from infected orchards and successfully isolated bacteriophages with potent lytic activity against the pathogen from the surrounding soil. We then characterized the biological and environmental stability, as well as the genomic features, of a particularly promising bacteriophage strain, revealing its potential for sustainable fire blight control in the region.

## 2. Materials and Methods

### 2.1. Materials

#### 2.1.1. Bacterial Strains and Culture Media

Fourteen *E. amylovora* strains were isolated from orchards in Xinjiang exhibiting typical symptoms of fire blight from 2016 to 2023. These strains were isolated using tissue isolation methods, subjected to pathogenicity testing, identification, and preservation (Table 1). The *P. syringae* pv. *syringae* strain D1, *B. velezensis* strains FN12, F34, F44, and the *B. amyloliquefaciens* strain FX74 were also isolated and stored in our laboratory. The *Escherichia coli* strain B13 was provided by the Agricultural Microbiology Laboratory at Xinjiang Agricultural University.

Nutrient Agar (NA) (g/L) (beef extract 3, sucrose 5, NaCl 5, peptone 10, agar 15) was used for bacterial culture. Soft agar (6 g/L) was used for a double-layer overlay plate.

#### 2.1.2. Soil Samples

Soil samples (134 in total) were collected from orchards in areas severely affected by fire blight in Korla City and Yuli County, Bazhou, Xinjiang. The samples were taken from the soil surface (5–15 cm) around diseased plants after removing surface debris.

### 2.2. Enrichment and Isolation

Five *E. amylovora* strains (*Ea* 001, FK-1, KRL-1, TC-1, and TC-2) were inoculated into NB broth. The cultures were shaken at 28 °C and 180 rpm for 24 h until the OD_600_ reached 0.8–1.0. The cultures were then diluted with sterile water to a concentration of 10^8^ CFU/mL, and equal volumes were mixed to prepare a composite bacterial solution.

Twenty grams of soil sample was added to 50 mL of the *E. amylovora* composite bacterial solution. After thorough mixing, the mixture was incubated overnight at 28 °C with shaking at 180 rpm. The culture was centrifuged at 10,000 rpm for 10 min, and the supernatant was filtered twice through a 0.22 μm microporous membrane (Navigator Lab Instrument, Tianjin, China) to obtain the filtrate for enrichment.

The double-layer agar plate method was employed for bacteriophage isolation [16]. One hundred microliters of the *E. amylovora* composite bacterial solution and 100 μL of the enrichment culture filtrate were mixed and incubated for 15 min. The mixture was evenly mixed with 7.5 mL of 45 °C NA soft agar and quickly poured onto a prepared NA plate. The solidified double-layer plates were inverted and incubated at 28 °C for 12 h until the plaque appeared.

### 2.3. Purification, Concentration, and Titer Determination

The bacteriophage plaques were picked up within 24 h and suspended in 1 mL of SM buffer (100 mM of NaCl, 8 mM of MgSO_4_, and 100 mM of Tris-HCl). After vortexing and crushing the plaques, the suspension was filtered twice through a 0.22 μm microporous membrane. Subsequently, the bacteriophage solution was serially diluted tenfold with SM buffer. A 100 μL aliquot of the diluted bacteriophage solution was mixed with 100 μL of the host bacterial suspension (OD_600_ = 0.5), incubated for 15 min, and then poured onto an NA plate. The plates were inverted and incubated at 28 °C until uniform plaque morphology was observed. This process was repeated 3–5 times until consistent plaque size and morphology were achieved.

The bacteriophage particles were precipitated by PEG8000 (100 g/L) from broth culture. The suspension was centrifuged at 10,000 rpm for 10 min at 4 °C. The pellet was resuspended in 2 mL of SM buffer and then filtered through a 0.22 μm filter to obtain the concentrated bacteriophage liquid.

The bacteriophage titer was determined by the double-layer overlay method. The titer (PFU/mL) was calculated using the formula: bacteriophage titer (PFU/mL) = (number of plaques/0.1 mL) × dilution factor.

### 2.4. Electron Microscopy

The bacteriophage was purified before undergoing negative staining with phosphotungstic acid. Subsequent observations and photographic documentation were carried out using a transmission electron microscope (TEM; model HT7800, Hitachi, Tokyo, Japan). The sizes of the bacteriophages were analyzed using Image-pro plus 6.0 software (Media Cybernetics, Inc., Rockville, MD, USA).

### 2.5. Characterization of Bacteriophage

#### 2.5.1. Determination of Optimal Multiplicity of Infection (MOI)

The host bacteria were cultured to the logarithmic phase (OD_600_ = 0.4~0.5). Different dilutions of bacteriophages with various MOI values (100, 10, 1, 0.1, 0.01, and 0.001) were mixed with an equal volume of host bacterial suspension. The mixture was incubated at room temperature for 15 min and then cultured on a shaker at 28 °C and 180 rpm for 9 h. The number of bacteriophage plaques was determined using the double-layer agar plate method. This process was repeated three times, and the bacteriophage titer under different MOI conditions was calculated.

#### 2.5.2. One-Step Growth Curve Determination

The method for the one-step growth curve experiment follows the approach described by Wen and Liang et al. [17,18]. Host bacterial cultures grown to the logarithmic phase (OD_600_ = 0.2~0.3, concentration of 2.05 × 10^7^ CFU/mL) were processed by taking a 5 mL sample and centrifuging at 10,000 rpm for 2 min. The supernatant was discarded, and the pellet was resuspended in 5 mL of NB medium; this process was repeated twice. Phage dilutions were added at an optimal multiplicity of infection (MOI) ratio of 1:1, thoroughly mixed, and incubated at room temperature for 10 min to allow for adsorption. The mixtures were then centrifuged to remove free phages in the supernatant. The pellet was resuspended in 10 mL of NB medium and incubated on a shaker at 28 °C with shaking at 180 rpm. Samples of 600 µL were taken every 10 min for a duration of 120 min from the start of the incubation. The collected samples were centrifuged at 10,000 rpm for 2 min and filtered through a 0.22 µm filter to obtain phage lysate, which was then diluted and mixed with host bacterial cultures to prepare double-layer agar plates for the determination of phage titers. The burst size was calculated using the formula: burst size = titer at the end of the burst period/initial concentration of host bacteria at the time of infection.

#### 2.5.3. Host Range

The spot method was used to determine the bacteriophage host range [19]. Fourteen strains of *E. amylovora*, one strain of *E. coli*, one strain of *P. syringae* pv. *syringae*, one strain of *B. amyloliquefaciens* and three strains of *B. velezensis* were used. The bacterial suspensions (OD_600_ of 0.8~1.0) were diluted to a concentration of 10^8^ CFU/mL with sterile water. A 100 μL aliquot of each bacterial suspension was mixed with 7.5 mL of NA soft agar medium and poured onto an NA plate to create double-layer agar plates. After solidification, 10 μL of bacteriophage solutions at different dilutions (10^0^–10^−7^) were spotted onto them. The plates were incubated at 28 °C overnight, and the formation of bacteriophage plaques was observed.

#### 2.5.4. Tolerance to Temperature, pH, UV Light, and Chemicals

Temperature: Bacteriophage lysate (10^9^ PFU/mL) was treated at 4 °C, 28 °C, 37 °C, 45 °C, 50 °C, 55 °C, 60 °C, 65 °C, and 70 °C in a water bath. Samples were taken at 0, 15, 30, 45, and 60 min. The treated bacteriophage lysate was mixed with an equal volume of *E. amylovora* liquid, and the bacteriophage titer was determined as above.

pH: Set NB medium at pH values of 3, 4, 5, 6, 7, 8, 9, 10, and 11. After incubating bacteriophage at 28 °C with shaking at 180 rpm for 2 h, the treated bacteriophage titer was determined as above.

UV tolerance: Bacteriophage (10^9^ PFU/mL) was placed in a 90 mm sterile Petri dish and exposed to UV light (30 W) at a distance of 30 cm for 30, 60, 90, 120, 150, and 180 min. The treated bacteriophage was determined as above.

Chemicals tolerance: CHCl_3_ (99%, Zhiyuan chemical reagent, Tianjin, China) and C_3_H_8_O (99.7% Zhiyuan chemical reagent, Tianjin, China), and H_2_O_2_ (20% Zhiyuan chemical reagent, Tianjin, China) and C_2_H_5_OH (99% Xinbote Chemical reagent, Tianjin, China) were added to the bacteriophage culture medium (10^9^ PFU/mL), resulting in a final concentration of 5%, 5%, 3%, and 75%, respectively. After incubating at 28 °C for 12 h, the bacteriophage titer was determined as above.

All of the above assays were repeated three times.

### 2.6. Whole Genome Sequencing

Bacteriophage RH-42-1 DNA was extracted using a phage DNA extraction kit (Magen Biotechnology, Guangzhou, China) according to the manual instructions. The DNA was sequenced via the Illumina NovaSeq platform at Personal Biotechnology Corp (Shanghai, China). The genome sequence was assembled using A5-MiSeq (v20160825) [20] and SPAdes (v3.12.0) [21]. Pilon software (v1.18) [22] was used for correction to obtain the final viral genome sequence. GeneMarkS (v4.2) [23] was employed to predict protein-coding genes in the genome, while Diamond (v0.8.36) [24] was used for sequence alignment and functional annotation of protein-coding genes. CGView [25] was utilized to create the genome circular map.

The full genome sequence, as well as the amino acid sequences of the bacteriophage terminase large subunit and capsid protein, were aligned with the GenBank database. Sequences with high homology were selected to construct a phylogenetic tree using MEGA11.0 software [26] based on the neighbor-joining method.

### 2.7. Statistical Analysis

The results were expressed in the form of mean ± standard deviation (SD) in appropriate places. Origin 2022 software was used to analyze the data.

## 3. Results and Analysis

### 3.1. Isolation, Purification, and Electron Microscopy

**Figure 1:**
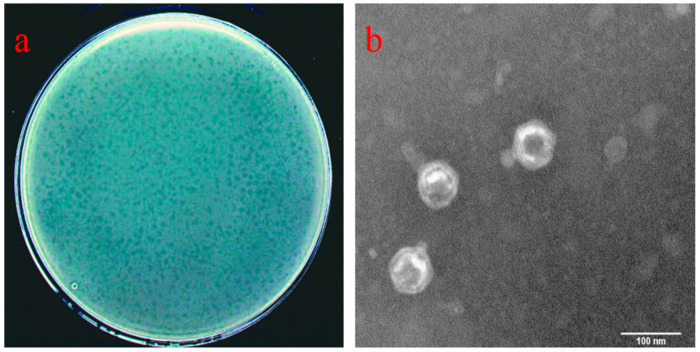
(**a**) Plaque morphology of RH-42-1 on *Erwinia amylovora* KER-1. Clear lysis plaques with a diameter of 3.29 ± 0.02 mm are evident (*n* = 30). (**b**) Transmission electron microscopy (TEM) image of RH-42-1 virions. The head diameter is 69.44 ± 1.96 nm, and the tail length is 21.13 ± 3.30 nm (*n* = 15). The scale bar represents 100 nm.

Among the 134 soil samples, 22 samples showed bacteriophage enrichment. After 10–12 h of incubation, plaques of various sizes developed on the bacterial lawns of *E. amylovora*. Two samples exhibited large and clear plaques, 12 samples had pinpoint-sized plaques, and 8 samples showed halo-like plaques around the colonies. Following repeated purification, two bacteriophages were obtained from the two samples with big and clear plaques. The plaques produced by these bacteriophages were circular, transparent, with smooth edges, had no halos, and were similar in size. One of them, with a plaque diameter of 3.29 ± 0.02 mm (*n* = 30), as shown in Figure 1a, had a titer of 9.6 × 10^9^ PFU/mL. It was assigned the name RH-42-1 (indicating that the strain was isolated from the 42nd soil sample of Renhe Farm, Xinjiang, China).

TEM revealed that bacteriophage RH-42-1 has a tadpole-like morphology with a clear icosahedral head, a diameter of 69.44 ± 1.96 nm (*n* = 15), and a non-contractile tail in length 21.13 ± 3.30 nm (*n* = 15), as shown in Figure 1b.

### 3.2. Optimal Multiplicity of Infection (MOI) for Bacteriophage RH-42-1

By co-culture different ratios of bacteriophages with the host bacteria and determining their infectivity, the results are presented in Table 2. When the MOI value was 1.0, the potency of bacteriophages was the highest, reaching 9.6 × 10^9^ PFU/mL. Therefore, the optimal MOI for this bacteriophage was determined to be 1:1.

### 3.3. One-Step Growth Curve of Bacteriophage RH-42-1

**Figure 2:**
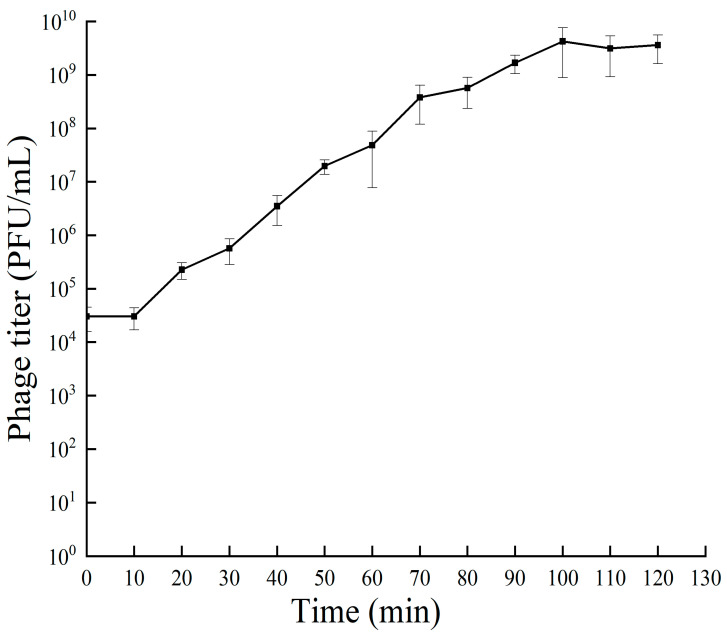
One-step growth curve of phage RH-42-1 on *Erwinia amylovora* KER-1 at different time points. Data points represent the mean of three independent experiments. The standard error of the mean is shown by error bars.

The results of the one-step growth curve for bacteriophage RH-42-1 are shown in Figure 2. Within the first 10 min of incubation, the number of phages remained constant in a latent stage. From 20 to 100 min post-infection, the quantity of RH-42-1 sharply increased, entering the burst phase in 80 min and a burst size of 2.07 × 10^2^ PFU/cell. After 100 min of incubation, the bacteriophage quantity stabilized, with a potency plateauing around 10^9^ PFU/mL.

### 3.4. Host Range of Bacteriophage RH-42-1

The host range of bacteriophage RH-42-1 was determined by testing its ability to lyse different strains, as shown in Table 3. RH-42-1 could lyse five different strains from different sources and hosts, including FK-1, KEL-1, KEL-2, TC-1, and TC-2. It did not exhibit lytic activity against *E. coli*, *P. syringae* pv. *syringae*, one strain of *B. amyloliquefaciens* and three strains of *B. velezensis* which are antagonistic to *E. amylovora*.

### 3.5. Tolerance of Bacteriophage RH-42-1

#### 3.5.1. Temperature Tolerance

**Figure 3:**
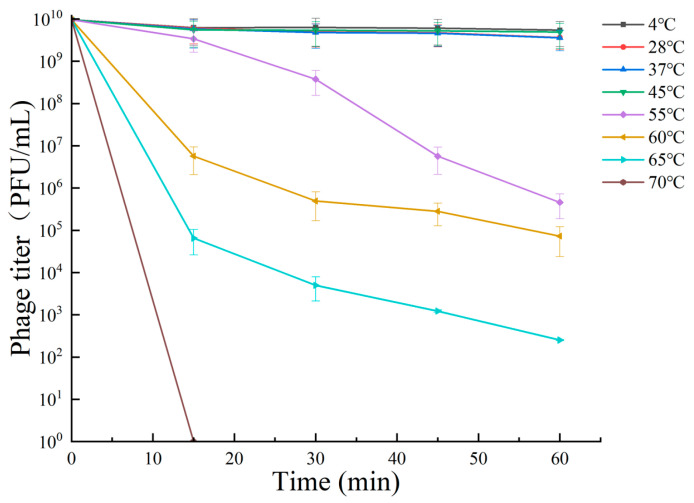
Tolerance of phage RH-42-1 to varying temperatures. The standard error of the mean is depicted by error bars.

The ability of bacteriophage RH-42-1 to lyse under different temperature conditions is shown in Figure 3. At temperatures ≤45 °C, RH-42-1 maintained high activity, and its potency remained relatively stable within the first 60 min of treatment. When the temperature was ≥55 °C, the activity of the bacteriophage started to decrease, and potency decreased with increasing treatment time. At 60 °C, after 60 min of treatment, the potency remained at 7.29 × 10^4^ PFU/mL. At temperatures ≥65 °C, the potency rapidly decreased, with a 7-log reduction after 60 min. At 70 °C, the activity was completely lost after 15 min of treatment.

#### 3.5.2. pH Tolerance

**Figure 4:**
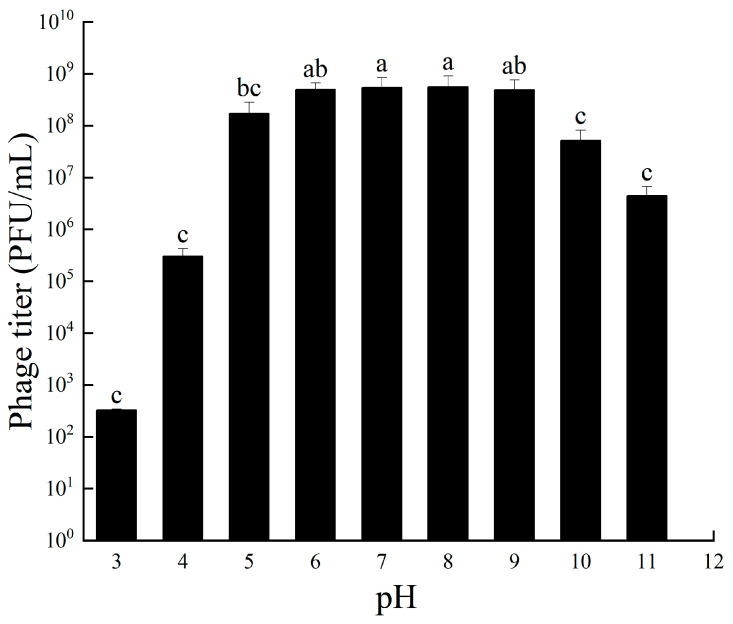
Tolerance of Phage RH-42-1 to Varied pH Levels. Error bars represent the standard error of the mean. The utilization of distinct letters (e.g., a, b, and c) denotes the presence of statistically significant differences (*p* < 0.05) within the observed data.

The potency of bacteriophage RH-42-1 under different pH conditions is depicted in Figure 4. Within the pH range of 5–9, RH-42-1 maintained high activity with stable potency. The highest potency (9.6 × 10^9^ PFU/mL) was observed at pH 7. Beyond this range, potency started to decrease. At pH 3–4, potency dropped by 4–6 logs, and at pH 10–11, potency decreased by 2–3 logs, still maintaining relatively high activity, demonstrating strong alkali tolerance.

#### 3.5.3. UV Radiation Tolerance

**Figure 5:**
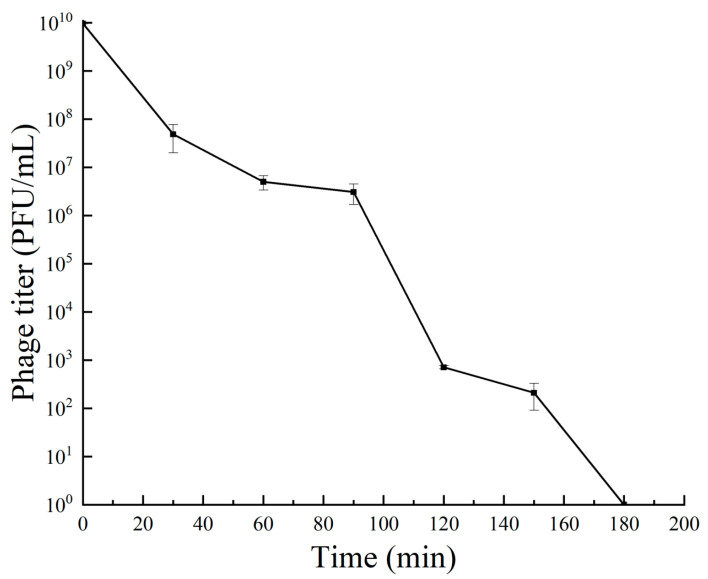
Phage RH-42-1 Tolerance to Ultraviolet Radiation. Error bars depict the standard error of the mean.

Bacteriophage RH-42-1 exhibited a gradual decrease in potency with prolonged UV radiation, as shown in Figure 5. After continuous exposure for 60 min, there was approximately a 2-log reduction, after 150 min, a 7-log reduction, and after 180 min, complete inactivation. This indicates that bacteriophage RH-42-1 has some tolerance to 60 min of UV radiation.

#### 3.5.4. Other Chemicals Tolerance

**Figure 6:**
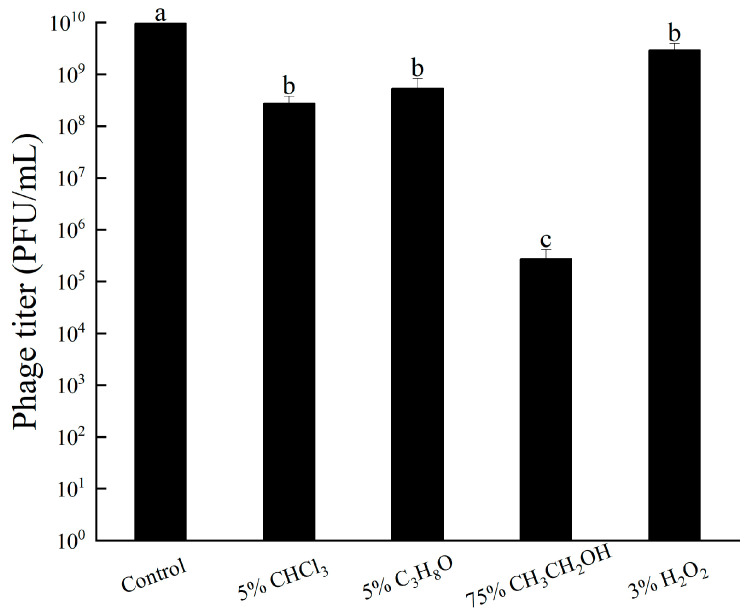
Effect of Different Chemicals on Phage RH-42-1 Viability. Standard error of the mean is shown by error bars. Consider specifying the tested chemicals for clarity. The utilization of distinct letters (e.g., a, b, c) denotes the presence of statistically significant differences (*p* < 0.05) within the observed data.

The results indicate that CHCl_3_, C_3_H_8_O, and 3% H_2_O_2_ had minimal impact on the lytic ability of bacteriophage RH-42-1, with a decrease of approximately 1 log in potency within 12 h, indicating strong tolerance (Figure 6). However, 75% ethanol significantly affected the bacteriophage, leading to a reduction of approximately 4 logs in potency.

### 3.6. Genomic Features of Bacteriophage RH-42-1

**Figure 7:**
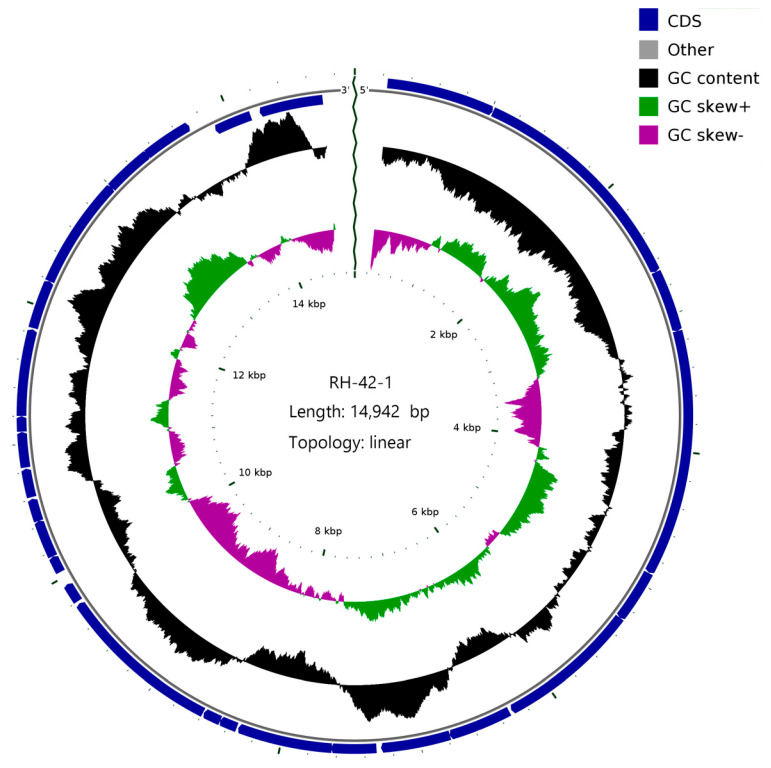
Comprehensive Genomic Map of Phage RH-42-1. (From interior to exterior): (1) Scale bar; (2) GC skew; (3) GC content; (4) positions of coding sequences (CDS), transfer RNA (tRNA), and ribosomal RNA (rRNA) of the genome.

The complete genome of bacteriophage RH-42-1 was sequenced and subjected to comparative genomic analysis. The results revealed that the genome of bacteriophage RH-42-1 is a linear double-stranded DNA molecule with a size of 14,942 bp and a GC content of 48.19% (NCBI accession number: PP099880). The genome map is presented in Figure 7. The genome contains 28 open reading frames (ORFs), out of which 25 have been functionally annotated, encoding structural proteins, DNA replication and regulatory proteins, perforin, and endolysin, among others. It possesses a holin–lysin system associated with bacterial lysis, including endolysin (accession number YP_338008.1) and holin (accession number AAX45661.1). Three ORFs were annotated as hypothetical proteins (Table 4). No tRNA genes were found, indicating the high dependence of bacteriophage RH-42-1 on the host’s translation machinery.

**Figure 8:**
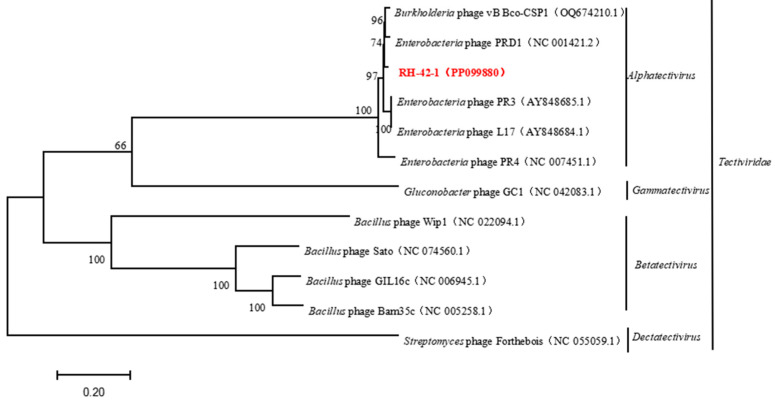
Phylogenetic Analysis of Phage RH-42-1 Based on its Complete Genome. The red text indicates the phage RH-42-1 isolated in this study.

The genome lacks antibiotic resistance genes and genes related to virulence factors, suggesting its suitability for safe applications without the risk of interspecies gene transfer. Comparative analysis of the complete genome sequence of bacteriophage RH-42-1 with the GenBank database and the construction of a phylogenetic tree (Figure 8) demonstrated that this bacteriophage clusters with the *Tectiviridae* family. It exhibits high homology (95.34%~97.55%) with *Tectiviridae*, *Alphatectivirus*, and five bacteriophages from the *Enterobacteria* phage family: PR3 (AY848685.1), L17 (AY848684.1), PRD1 (NC001421.2), PR4 (NC007451.1), and the *Burkholderia* phage vB_Bco-CSP1 (OQ674210.1), placing them in the same evolutionary branch.

**Figure 9:**
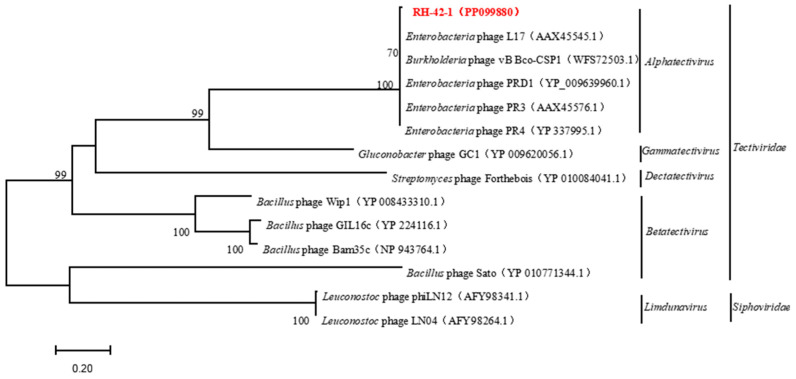
Phylogenetic Relationship of Phage RH-42-1 Based on Its Major Capsid Protein Sequence. The red text indicates the phage RH-42-1 isolated in this study.

**Figure 10:**
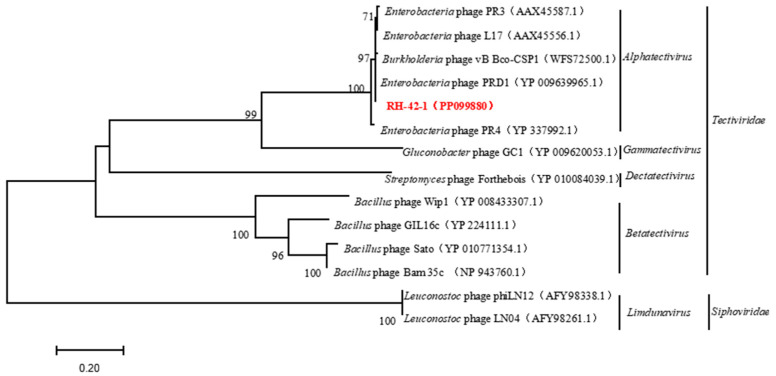
Phylogenetic Relationship of Phage RH-42-1 Determined by Its Terminase Large Subunit Sequence. The red text indicates the phage RH-42-1 isolated in this study.

Furthermore, the conserved protein-coding genes, terminase large subunit, and major capsid protein of bacteriophage RH-42-1 were compared with others in the GenBank database. The amino acid sequence of the major capsid protein of RH-42-1 showed high homology (99.49–99.56%) with five bacteriophages from the database (Figure 9). The amino acid sequence of the terminase large subunit exhibited high homology (98.24–99.56%) with four bacteriophages from the database (Figure 10). The phylogenetic tree, construction based on these highly similar bacteriophages, consistently placed RH-42-1 within the *Tectiviridae* family, *Alphatectivirus* genus, with the closest relationship to *Enterobacteria* phage PRD1 (NC 001421.2).

In conclusion, bacteriophage RH-42-1 is identified as a member of the *Tectiviridae* family, *Alphatectivirus* genus.

## 4. Discussion

Numerous studies have highlighted the strong host specificity, rapid replication, and potent antibacterial properties of lytic bacteriophages. They show promising applications in the safe and effective control of pathogenic bacteria in both plants and animals, addressing issues related to bacterial resistance and rapid detection [27]. Therefore, the identification of bacteriophages with strong lytic activity against target pathogenic bacteria and high stability is crucial for the development of bacteriophage-based biocontrol agents. The key to isolating virulent bacteriophages lies in the collection, enrichment, and purification of environmental samples containing bacteriophages. In this study, using *E. amylovora* strains isolated from Xinjiang as the target host bacteria, bacteriophages were isolated from 134 soil samples collected around severely affected fire blight orchards. Only 22 samples yielded bacteriophage plaques, and among them, 20 samples had very small plaques that could not be consistently formed during purification, indicating the possible presence of weak or temperate bacteriophages. Two samples produced large and clear plaques, from which two bacteriophages were purified. Among them, bacteriophage RH-42-1 produced plaques that were large, uniform, transparent, with high efficacy and stability, exhibiting the typical characteristics of lytic bacteriophages.

The fire blight pathogen exists in ulcerous lesions on branches, diseased branches, leaves, fruits, orchard soil, and irrigation water [28]. Although efforts have been made in the preliminary stages of this study to isolate bacteriophages from materials such as bark, branches, and soil from orchards with fire blight, only soil samples yielded bacteriophages. Similarly, Meczker et al. [29] isolated bacteriophages belonging to the *Siphoviridae* family from soil samples collected from apple orchards affected by fire blight. They suggested that orchard soil during disease outbreaks could be a suitable source for selecting bacteriophages against the target bacteria. This finding aligns with the results of the current study.

Compared with phage isolation from the soil of an *Xanthomonas*-infected rice paddy, the current study had a lower probability of isolating virulent bacteriophages against fire blight bacteria [30]. This difference might be attributed to the use of a mixture of five strains of fire blight bacteria as the target host in this study. Due to the strong specificity of bacteriophages, the selection for infection of different strains within the fire blight bacteria might have led to the failure of some bacteriophages with strong specificity that could not infect the mixed host strains to be isolated. This result suggests that, when isolating lytic bacteriophages, it is advisable to mix multiple strains from different sources as target host bacteria, which can increase the success rate of isolation.

A bacteriophage’s host range is closely related to the source of its isolation material. Balogh et al. [31] isolated bacteriophages against *X. axonopodis* pv. *citri* from citrus canker lesions and found that the host range was narrow. In contrast, bacteriophages isolated from soil and sewage had a broader host range. Bacteriophage RH-42-1, isolated from soil, could lyse five different strains of fire blight bacteria from different sources and hosts but could not lyse the nine other strains of fire blight bacteria tested. Additionally, it did not lyse one strain of *E. coli*, one strain of *P. syringae*, one strain of *B. amyloliquefaciens* and three strains of *B. velezensis*, preliminarily indicating its host specificity.

This phenomenon was also observed by Natalya V et al. [32] and Akremi et al. [14] who identified the fire blight bacteriophages Loshitsa2 and Micant from soil, which can lyse six strains of the pathogen *E. amylovora* but cannot lyse the others. Akremi et al. [14] isolated the fire blight bacteriophage PEar6 from soil, which can lyse eight strains of the fire blight pathogen, but not for others. It has been reported that bacteriophages isolated from environmental samples with low host density have a wider host range than those from samples with high host density [33]. To obtain bacteriophages with a broader spectrum and wider applications, future work may consider collecting samples from orchards with mild occurrences of fire blight or healthy orchards for bacteriophage isolation.

Different bacteriophages have different latent periods, burst times, and burst sizes. Kim et al. [34] isolated the fire blight bacteriophage pEp_SNUABM_08, which has a latent period of 40 min and a burst size of 20 phages. Akremi et al. [14] isolated the fire blight bacteriophage PEar 6, which has a latent period of 20 min, a rising burst period of 25 min, and a burst size of about 280 PFU/cell. Compared with the above fire blight bacteriophages, PEar 6 belongs to a bacteriophage category with short latent periods and long burst times. This indicates that this bacteriophage has strong replication activity and can invade and lyse the host bacteria in a short time.

The activity of bacteriophages sprayed onto the surface of plants can be affected by various environmental factors in real-world applications, which directly affects the bactericidal effect. Understanding the adaptability of bacteriophages to environmental factors is the basis for evaluating their application potential in biocontrol, and it also provides a reference for the subsequent research and development of bacteriophage preparations and application technology. Those phages have with variety of characteristics. Park et al. [35] isolated the fire blight bacteriophage PhiEap-8, which exhibited tolerance to temperatures up to 50 °C, pH ranging from 4 to 11, and 60 min of UV irradiation. Jo et al. [36] isolated the fire blight bacteriophage pEp_SNUABM_03, which showed tolerance to temperatures up to 40 °C and pH levels between 4 and 9. Choe et al. [37] isolated the fire blight bacteriophage ΦFifi106, which maintained certain activity at temperatures up to 50 °C, pH levels between 4 and 11, and under 6 h of UV irradiation.

The bacteriophage RH-42-1 maintained a titer of 7.29 × 10^4^ PFU/mL after being treated at 60 °C for 60 min, showing a high temperature resistance. The bacteriophage has a strong resistance to an alkaline environment and can still maintain high activity in a strongly alkaline environment of pH 10–11. The bacteriophage RH-42-1 has a certain tolerance to short-term (60 min) ultraviolet radiation. It exhibited a certain level of resistance to 5% chloroform, 5% isopropanol, and 3% hydrogen peroxide, but sensitivity to 75% ethanol.

Regarding the morphology of the fire blight pathogen phages. Muller et al. [38] isolated the fire blight bacteriophages φEa1 and φEa100, which, respectively, exhibited icosahedral heads with diameters of 59.89 ± 1.49 nm and 61.42 ± 2.14 nm, both belonging to the *Podoviridae* family. φEa116 possessed an icosahedral head with a diameter of 73.036 ± 1.89 nm and a non-contractile tail measuring 114.42 ± 2.51 nm in length, classified under the *Myoviridae* family. Miloud Sabri et al. [39] isolated the fire blight bacteriophage IT22, featuring an icosahedral head measuring 90.00 ± 5.00 nm in length and 75.00 ± 3.00 nm in width, with a contracted tail length of 100.00 ± 10.00 nm, belonging to the *Inoviridae* family. Through electron microscopy observation, the bacteriophage RH-42-1 isolated in this study exhibited a tadpole-like morphology, with an icosahedral head diameter of 69.44 ± 1.96 nm and a short, non-contractile tail measuring 21.13 ± 3.30 nm in length. Combining whole-genome sequencing and conservative protein functional gene phylogenetic analysis, the fire blight bacteriophage RH-42-1 was identified as belonging to the genus *Alphatectivirus* of the family *Tectiviridae*.

The currently reported fire blight bacteriophages are mainly classified into *Podoviridae* [38], *Myoviridae* [40], and *Inoviridae* [39]. However, through TEM, genome sequencing, and conservative protein gene analysis, the bacteriophage RH-42-1 was identified as a member of the *Tectiviridae* family, *Alphatectivirus* genus. The reported hosts of *Tectiviridae* bacteriophages include *P. fluorescens* [41], *Bacillus* [42], *Streptomyces*, and *Gluconobacter* [43]. There is no host report on *E. amylovora*. The bacteriophage RH-42-1 isolated in this study is the first report of *E. amylovora* as the host bacterium of a *Tectiviridae* bacteriophage.

Whole-genome sequencing and phylogenetic analysis of conserved protein functional genes revealed that the fire blight pathogen phage RH-42-1 belongs to the family *Tectiviridae*, genus *Alphatectivirus*. Comparison with the reference PRD1 phage from the International Committee on Taxonomy of Viruses (ICTV) database showed several key differences. Unlike PRD1, which has 13 genes located on the negative strand, RH-42-1 has only 2 genes on the negative strand, with the remaining genes on the positive strand. Additionally, nine proteins encoded by RH-42-1 displayed amino acid sequence variations compared to their PRD1 counterparts.

Additionally, differences exist between them in certain characteristics. Bamford et al. [44] reported that PRD1 bacteriophages isolated from sewage are sensitive to chloroform, whereas RH-42-1 bacteriophages isolated in this study from soil exhibit tolerance to chloroform. PRD1 bacteriophages infect host bacteria of the *pseudomonads* and Enterobacteriaceae families containing P or W incompatible plasmids and rely on host plasmids for replication [45]; whereas, RH-42-1 infects host bacteria of the fire blight bacterium which commonly contain the PEA29 plasmid, and some strains contain plasmids such as PEA68, PEA34, PEA72, and PEA8.7 [46]. However, whether RH-42-1 relies on host bacterial plasmids for replication and whether the host bacteria contain incompatible group plasmids require further investigation and confirmation.

Whole genome sequencing analysis showed that the bacteriophage RH-42-1 has lysozyme and perforin, but does not contain integrase, transposase, antibiotic resistance genes, and virulence factors, which supports the notion that the bacteriophage belongs to the range of lytic bacteriophages that can be safely applied.

## 5. Conclusions

This study successfully isolated a lytic bacteriophage, RH-42-1, against *E. amylovora*, the causal agent of fire blight, from disease-affected areas in Xinjiang, China. Through characterization and genomic analysis, bacteriophage RH-42-1 was identified as a member of the *Tectiviridae* family, specifically belonging to the *Alphatectivirus* genus. Notably, this marks the first report of a *Tectiviridae* bacteriophage infecting *E. amylovora*.

Compared to other reported lytic bacteriophages against *E. amylovora*, RH-42-1 exhibited unique characteristics. In conclusion, bacteriophage RH-42-1, isolated from fire blight affected areas in Xinjiang, China, represents a promising candidate for the biological control of *E. amylovora*. Its unique biological characteristics, strong lytic activity, and environmental resilience make it a valuable resource for future studies and the development of bacteriophage-based biocontrol agents.

## Figures and Tables

**Table 1:** *Erwinia amylovora* Strains Employed in this Investigation.

Strain Number	Origin Tissues	Places
Ea 001	Apple branches	Khorgas city, Xinjiang
X24	Fragrant pear leaf	Korla city, Xinjing
X26	Fragrant pear branch	Bugur county, Xinjing
X36	Fragrant pear leaf	Korla city, Xinjing
Y85	Fragrant pear core	Korla city, Xinjing
Y126	Hawthorn leaf	Bugur county, Xinjing
Y134	Quince branch	Bugur county, Xinjing
Y137	Fragrant pear branch	Korla city, Xinjing
FK-1	Apple branches	Fukang city, Xinjing
KEL-1	Fragrant pear leaf	Yuli county, Xinjing
KEL-2	Fragrant pear branch	Yuli county, Xinjing
TC-1	Apple branches	Tacheng city, Xinjing
TC-2	Apple branches	Tacheng city, Xinjing
TC-3	Hawthorn branch	Tacheng city, Xinjing

**Table 2:** Identifying the Optimal Multiplicity of Infection (MOI) for Phage RH-42-1.

MOI	Bacterial Population (CFU/mL)	Phage Concentration (PFU/mL)	Phage Titer (PFU/mL)
100:1	10^7^	10^9^	(2.7 ± 0.15) × 10^7^
10:1	10^7^	10^8^	(4.4 ± 0.2) × 10^7^
1:1	10^7^	10^7^	(9.6 ± 0.1) × 10^9^
0.1:1	10^7^	10^6^	(1.54 ± 0.03) × 10^8^
0.01:1	10^7^	10^5^	(6.26 ± 0.15) × 10^7^
0.001:1	10^7^	10^4^	(4.18 ± 0.03) × 10^6^

**Table 3:** Host Range Determination of Phage RH-42-1: Lytic or Non-lytic Activity.

Host Species	Strains	Lysis Result
*E*. *amylovora*	FK-1, KEL-1, KEL-2, TC-1, TC-2	+
	Ea001, TC-3, X24, X26, X36, Y85, Y126, Y134, Y137	−
*P*. *syringae* pv. *syringage*	D1	−
*Escherichia coli*	B13	−
*B*. *velezensis*	FN12, F34, F44	−
*B*. *amyloliquefaciens*	FX74	−

“+” indicates that it can be cleaved by phages, and “−” indicates that it cannot be lysed.

**Table 4:** Open Reading Frame (ORF) Analysis of the RH-42-1 Phage Genome.

Functional Category	ORF Order	Position of ORF	Accession No.	Annotation
Structure and packaging	5	4908−5288	YP_009639959.1	major spike protein
	6	5288−6310	AAX45552.1	spike
	7	6329−6589	YP_009639961.1	protein P17
	8	6579−6785	AAR99748.1	protein P33
	9	6785−7285	YP_337990.1	minor head protein
	10	7318−7641	UDY80295.1	protein P10
	11	7638−8321	YP_009639965.1	terminase large subunit
	13	8461−8589	YP_009639967.1	packaging protein
	14	8596−9783	YP_337995.1	major capsid protein
	20	10,834−11,088	YP_009639975.1	minor capsid protein
DNA replication and regulation	1	234−1013	YP_009639955.1	terminal protein
	2	1017−2678	AAR99740.1	DNA polymerase
	4	3129−4904	AAX45635.1	receptor binding
	15	9802−9945	YP_337996.1	DNA packaging
	17	10,169−10,441	YP_009639972.1	DNA delivery
	18	10,441−10,605	YP_009639973.1	membrane DNA delivery
	19	10,618−10,824	YP_009639974.1	membrane DNA delivery
	22	11,203−11,826	YP_009639977.1	DNA delivery
	23	11,837−12,190	AAR99763.1	infectivity protein
	24	12,191−13,000	AAX45554.1	transclycosylase
	26	13,343−13,705	YP_009639982.1	hypothetical protein
	27	13,861−14,145	AAR99769.1	ssDNA binding protein
	28	14,218−14,700	YP_009639985.1	single strand DNA binding protein
Cracking	3	2680−3129	YP_338008.1	endolysin
	25	12,997−13,350	AAX45661.1	holin
Unknown	12	8333−8461	YP_009639966.1	hypothetical protein
	16	10,045−10,167	YP_009639971.1	hypothetical protein
	21	11,091−11,201	YP_009639976.1	hypothetical protein

## Data Availability

The sequencing data are available from NCBI GenBank under accession number PP099880.
